# Impact of setup errors on multi‐isocenter volumetric modulated arc therapy for craniospinal irradiation

**DOI:** 10.1002/acm2.13044

**Published:** 2020-10-18

**Authors:** Yongqiang Zhou, Yao Ai, Ce Han, Xiaomin Zheng, Jinling Yi, Congying Xie, Xiance Jin

**Affiliations:** ^1^ Department of radiation and medical oncology the First Affiliated Hospital of Wenzhou Medical University Wenzhou China; ^2^ Department of radiation and medical oncology the Second Affiliated Hospital of Wenzhou Medical University Wenzhou China

**Keywords:** craniospinal irradiation, dosimetric measurement, setup errors, volumetric modulated arc therapy

## Abstract

Multi‐isocenter volumetric modulated arc therapy (VMAT) is recommended for craniospinal irradiation (CSI) to smooth the dose distribution in the junction regions relying solely on inverse optimization. However, few studies have measured the dosimetric impact of setup errors on this multi‐isocenter VMAT in the junction areas. The purpose of this study is to evaluate the impact of positional errors during VMAT CSI with two‐dimension (2D) and three‐dimension (3D) dosimetric measurements. A total of 20 patients treated by three‐isocenter VMAT CSI were retrospectively reviewed and analyzed. A 3D diode array ArcCHECK and radiochromic film EBT3 were applied to measure the percentage gamma passing rates (%GPs) and dose distributions in the junction areas between the cranial/upper‐spinal and the upper/lower‐spinal fields with intentionally introduced setup errors of ± 1 mm, ±2 mm, ±3 mm, ±5 mm, and ± 8 mm, respectively. The length and volume of planning target volume (PTV) for these CSI patients ranged from 50.14 to 80.8 cm, and 1572.3 to 2114.5 cm^3^, respectively. The %GPs for ±3 mm, ±5 mm, and ±8 mm positional errors were around 95%, 90%, and 85%, respectively, in the junction areas. The dosimetric verification results with EBT3 films indicated that cold and hot areas were observed with the increase of introduced setup errors. In conclusion, the dosimetric verification with intentionally introduced setup errors demonstrated that positional errors within 3 mm have a little impact for VMAT CSI, although setup errors should be minimized. Relying on the inverse optimization of VMAT to smooth the dose distribution in the junction areas is feasible for CSI.

## 1 Introduction

Craniospinal irradiation (CSI) is a complex radiotherapy technique indicated for patients with high risk of whole central nervous system involvement.^1^, ^2^ The challenge of CSI is the exceeding length of planning target volume (PTV) over the maximum treatment field of a common linear accelerator (linac), which requires combining treatment fields to cover the whole targets and causes over‐ and/or underdose problems in the junction areas between fields.^3^, ^4^ Traditionally, 3D conformal radiotherapy (CRT) technique is applied for CSI by using two lateral opposed photon beams for the brain and matching to one or more posterior photon beams for the spine.^5^ Weekly junction displacements, known as feathering by moving the treatment field junction weekly, have been adopted to reduce the larger over‐ or underdose in the junction areas.^5^ However, junction issue remains challenging for 3DCRT due to the intrinsic limitations of the dose calculation accuracy, collimator positioning accuracy, patient positioning accuracy, etc.^3^, ^6^

In order to deliver more conformal doses to the target volumes and to better spare the surrounding health tissues outside of the target, in particular the thyroid, heart, and intestines,^7^ modern techniques, such as intensity‐modulated radiation therapy (IMRT),^8^ volumetric modulated arc therapy (VMAT),^9^ tomotherapy,^10^ and proton therapy have been intensively investigated in the dose delivery of CSI.^11^ Studies demonstrated superior dosimetric results from these modern techniques in comparison with conventional 3DCRT for CSI; however, there is still no consensus on the recommendation of CSI radiotherapy technique due to a larger number of organs at risk involved and overlap among different techniques and studies.^8^, ^9^, ^10^, ^11^, ^12^ Dosimetric hot and cold spots in the field junction regions are still a major concern. A variety of techniques, such as “jagged‐junction”, “overlap” technique, and “gradient optimization” technique, have been employed for these modern techniques to address the dosimetric problem in the field junctions.^5^, ^8^, ^10^, ^13^

Due to its advantage of less treatment time and potential decrease of movement uncertainty, especially for pediatric patients, linac‐based VMAT CSI has been investigated and applied in clinical practice. Controlling linear dose gradient across the junction,^14^ silico ideal‐based universal field matching solution,^15^ low gradient junction technique,^16^ etc., have been investigated specifically for VMAT CSI to optimize the junction doses. However, as an inversely optimized technique, careful planning of field junctions may complicate the VMAT planning process.^17^ Reports demonstrated that it is feasible to optimize a set of overlapping fields concurrently without explicitly controlling the junction dose with VMAT.^18^, ^19^ A smooth dose across the junction could be achieved with this simple approach by relying on the optimization algorithm. One limitation of this simple approach is that it renders the dosimetric distribution sensitive to positional errors. Myers et al. have calculated the possible impact of positional errors by mimicking the setup errors.^20^ However, few studies have measured the dosimetric impact of setup errors for VMAT CSI. The purpose of this study is to evaluate the dosimetric impact of positional errors during VMAT CSI with 2D and 3D quality assurance methods.

## 2 Materials and methods

### 2.A Patients and treatment planning

Patients treated with VMAT CSI at the author's hospital from December 2015 to December 2018 were retrospectively reviewed and analyzed in this study. All the patients were immobilized with a thermoplastic mask for head and neck in the prone position. The spine position was aligned with laser and checked with X‐ray topography for suitable tilt adjustment before CT simulation. The simulation was performed by a Philips Brilliant spiral CT (Philips Brilliant, Cleveland, OH, USA). The clinical target volume (CTV) was contoured by senior radiation oncologists by including the entire brain, meninges, and the spinal canal.^7^ The brain PTV was uniform expanded by 3 mm from CTV, and the spinal PTV was expanded by 5 mm from CTV.

**Fig. 1:**
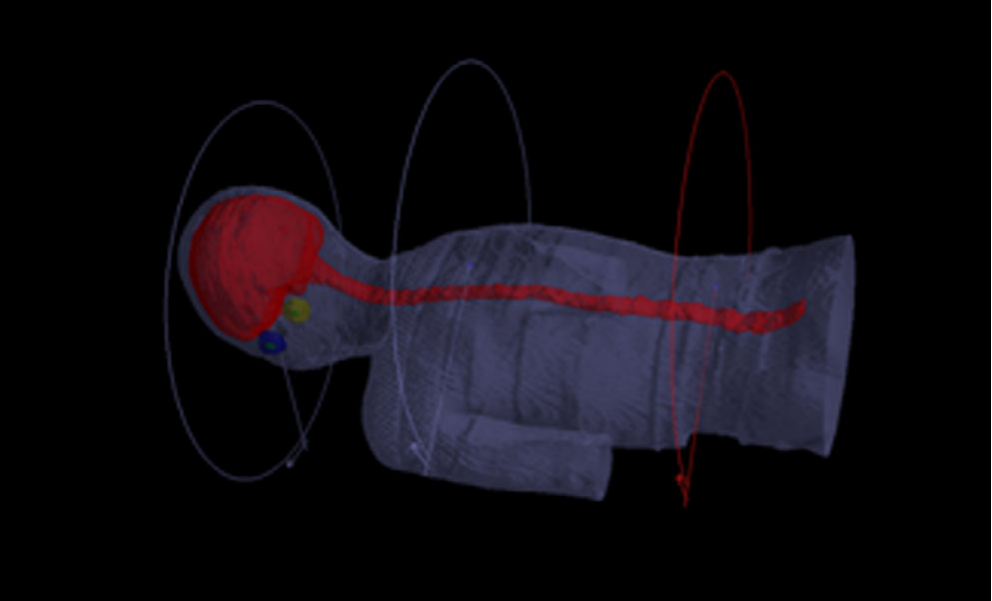
A typical planning target volume and beam arrangements for craniospinal irradiation with volumetric modulated arc therapy.

All VMAT CSI plans were created with three isocenters that were placed in the cranial, upper‐spinal (T3), and lower‐spinal (L2) regions. The separation between the cranial and the upper‐spinal isocenters ranged from 20 to 26 cm, and the separation between the upper and lower‐spinal isocenters ranged from 25 to 30 cm for these patients. The coordinates of the three isocenters differed only in the craniocaudal direction to simplify the positioning procedure. Two full arcs (clockwise and counterclockwise rotation) were used to cover the entire brain. Two arcs of 240° with clockwise rotation were used to cover the spinal cord. The collimator angles were set at 5° for the clockwise arc and 355° for the counter clockwise arc in order to minimize the tongue and grove effect of the multi‐leaf collimator (MLC).^21^ Approximately 6 cm overlap between adjacent fields was ensured to avoid matching junctions, and three overlapping arcs (four arcs) were optimized at the same time. A prescription dose of 27.2–30.6 Gy at 17 fractions was assigned to cover the 95% of the PTV. At least 95% PTV must be covered by 95% of the prescription dose. OAR constraints included brainstem, spinal cord, kidneys, and lung. A typical PTV and beam arrangement were presented in Fig. 1.

Treatment planning was optimized with Monte Carlo algorithm on Monaco 5.11 treatment planning system (Clinical version 5.1.1, Elekta, UK) for a 6‐MV photon beam with 3 mm × 3 mm × 3 mm calculation grid size. Dosimetric verifications were delivered though a MosaiQ® record and verify system v. 1.60Q3 (IMPAC Medical Systems, Inc., Sunnyvale, CA) at an Elekta Synergy linac equipped with an 80‐leaf MLC (MLCi2TM, Elekta Ltd, Crawley, UK).

### 2.B Dosimetric verification with ArcCHECK

**Fig. 2:**
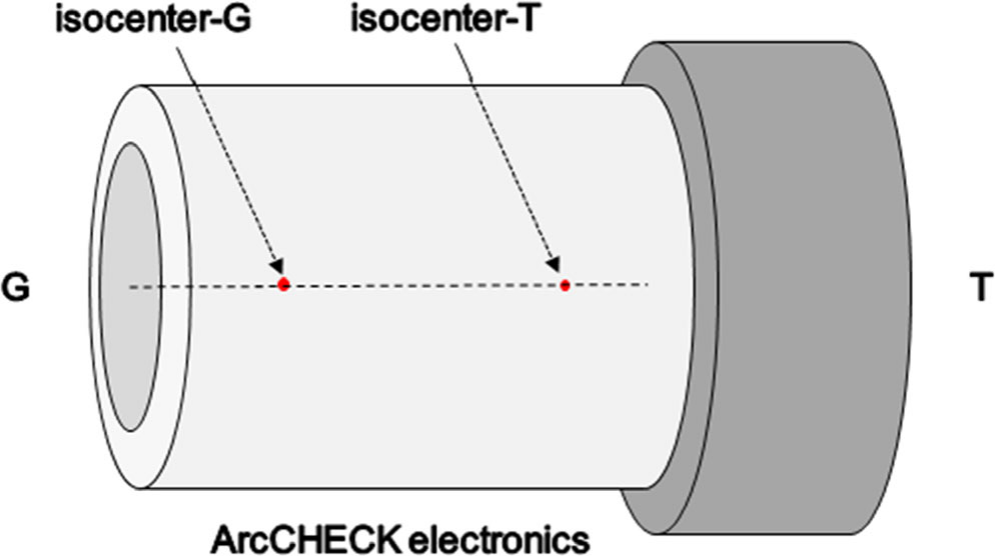
The experimental setup for the ArcCHECK phantom during 3D dosimetric verification, G = gun direction, T = target direction.

Three‐dimensional dosimetric verification of VMAT CSI plans was carried out by using a 3D diode array ArcCHECK (Model 1220) and SNC Patient software (v.6.2.1; Sun Nuclear Corporation). Due to the limited length of ArcCHECK phantom, dosimetric measurements in the cranial/upper‐spinal and the upper/lower‐spinal overlap regions were measured by shifting the couch in the caudal direction by 13 cm to account the cranial and upper‐spinal isocenters. RTplan, RTstructures, and RTdose files were exported from TPS to 3DVH program, and an ArcCHECK movie (ACML) file generated by the SNC Patient software was used for further 3D dosimetric difference analysis. A typical measurement phantom setup was presented in Fig. 2.

In order to measure the dosimetric impact of positional errors in the overlap regions, positioning errors were intentionally introduced by moving the isocenters of the VMAT fields. For the cranial/upper‐spinal field junction, the isocenter of the cranial VMAT fields was set correctly, then the isocenter of the upper‐spinal VMAT fields was moved to introduce positioning errors of ± 1 mm, ±2 mm, ±3 mm, ±5 mm, and ± 8 mm in the cranial or caudal direction, respectively. For upper/lower‐spinal field junction, the isocenter of the upper‐spinal VMAT fields was set correctly, then the isocenter of the lower‐spinal VMAT fields was moved to introduce positioning errors of ± 1 mm, ±2 mm, ±3 mm, ±5 mm, and ± 8 mm in the cranial or caudal direction, respectively. The similar procedure was carried out for the positional shifts of ± 1 mm, ±2 mm, and ± 3 mm in the lateral direction and anterior/posterior direction. In this research, we focus on the dosimetric impact of positional errors in the craniocaudal direction, because the positional errors in the craniocaudal direction can result in over‐ or underdosing in the field junction area. A gamma passing criteria of 3%/3 mm with a 10% lower dose threshold (TH) were applied for the quality assurance analysis.

### 2.C Dosimetric verification with radiochromic films

**Fig. 3:**
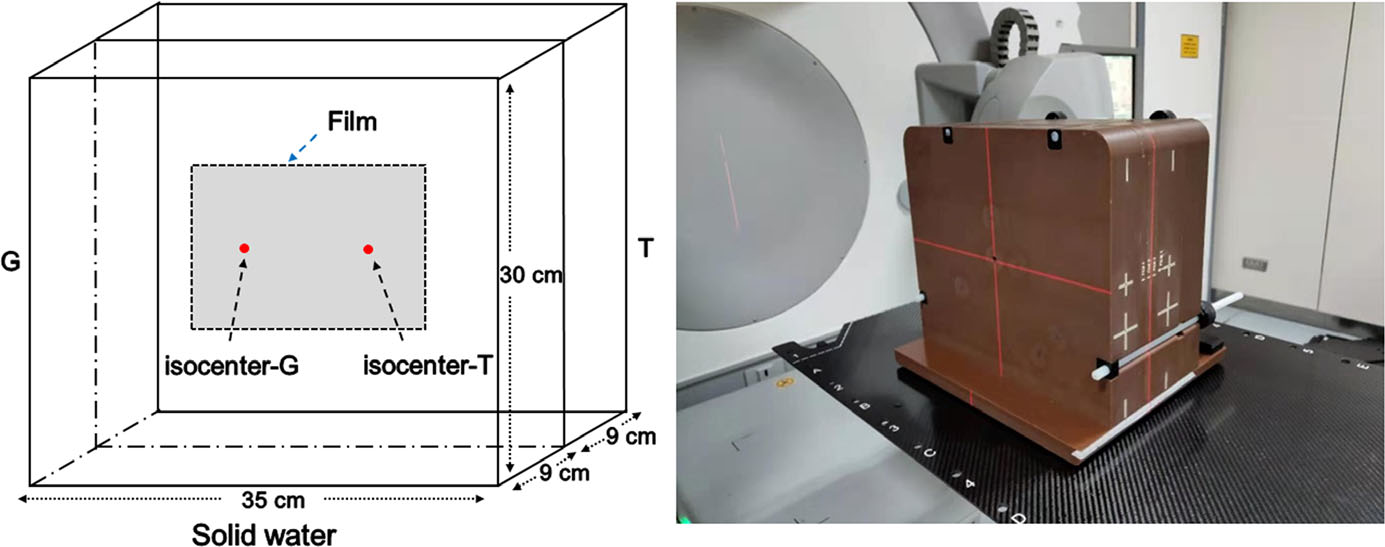
Phantom setup and schematic diagram for dosimetric verification with EBT3 film, G = gun direction, T = target direction.

The dosimetric verification was further validated by a radiochromic film‐ GafChromic EBT3 (International Specialty Products, Wayne, NJ, USA) along with a Benchmark phantom (Med‐Tec, Orange City, IA). EBT3 samples were clipped in the middle of the 18 cm thick phantom, whose middle was aligned with the junction regions. The positional errors were intentionally introduced in the longitudinal direction by ± 3 mm, ±5 mm, and ± 8 mm, respectively. A typical setup and schematic diagram for EBT3 measurement are shown in Fig. 3. The films were scanned and analyzed with an EPSON Perfection V750 (Seiko Epson Corporation, Nagano, Japan) desktop flat‐bed scanner.

## 3 Results

A total of 20 patients (3 females and 17 males) treated by VMAT CSI from December 2015 to December 2018 were enrolled in this dosimetric verification study. There were 16 patients diagnosed with medulloblastoma and four with pineal tumor at a mean age of 17 yr old (range from 4 to 47 yr). The length and volume of PTV range from 50.14 to 80.8 cm and 1572.3 to 2114.5 cm^3^, respectively. Detailed characteristics of these patients are presented in Table 1.

**Table 1:** Characteristics of enrolled patients treated with craniospinal irradiation with volumetric modulated arc therapy.

Patients	Pathology	Sex	Age (Year)	Length of PTV (cm)	Volume of PTV (cm^3^)
1	Medulloblastoma	Male	14	65.07	1877.6
2	Medulloblastoma	Male	4	50.14	1572.3
3	Medulloblastoma	Male	7	61.28	1818.4
4	Medulloblastoma	Male	32	78.15	1957.2
5	Medulloblastoma	Female	6	52.5	1696.9
6	Medulloblastoma	Male	10	61.6	1735.9
7	Medulloblastoma	Male	12	64	1894.9
8	Medulloblastoma	Male	16	80.8	1985.1
9	Medulloblastoma	Male	15	76.9	1929.5
10	Medulloblastoma	Male	10	61.8	2015.5
11	Medulloblastoma	Male	31	75.8	1917.9
12	Medulloblastoma	Male	6	56.1	1840.8
13	Medulloblastoma	Male	12	71.4	2113.9
14	Medulloblastoma	Female	47	80.4	2114.5
15	Medulloblastoma	Male	7	65.2	1856.2
16	Medulloblastoma	Male	21	71.4	1881
17	Pineal tumor	Female	18	76.5	1914.2
18	Pineal tumor	Male	4	53.2	1586.3
19	Pineal tumor	Male	33	73.9	1883.9
20	Pineal tumor	Male	42	80.4	1983.2

PTV, planning target volume.

**Fig. 4:**
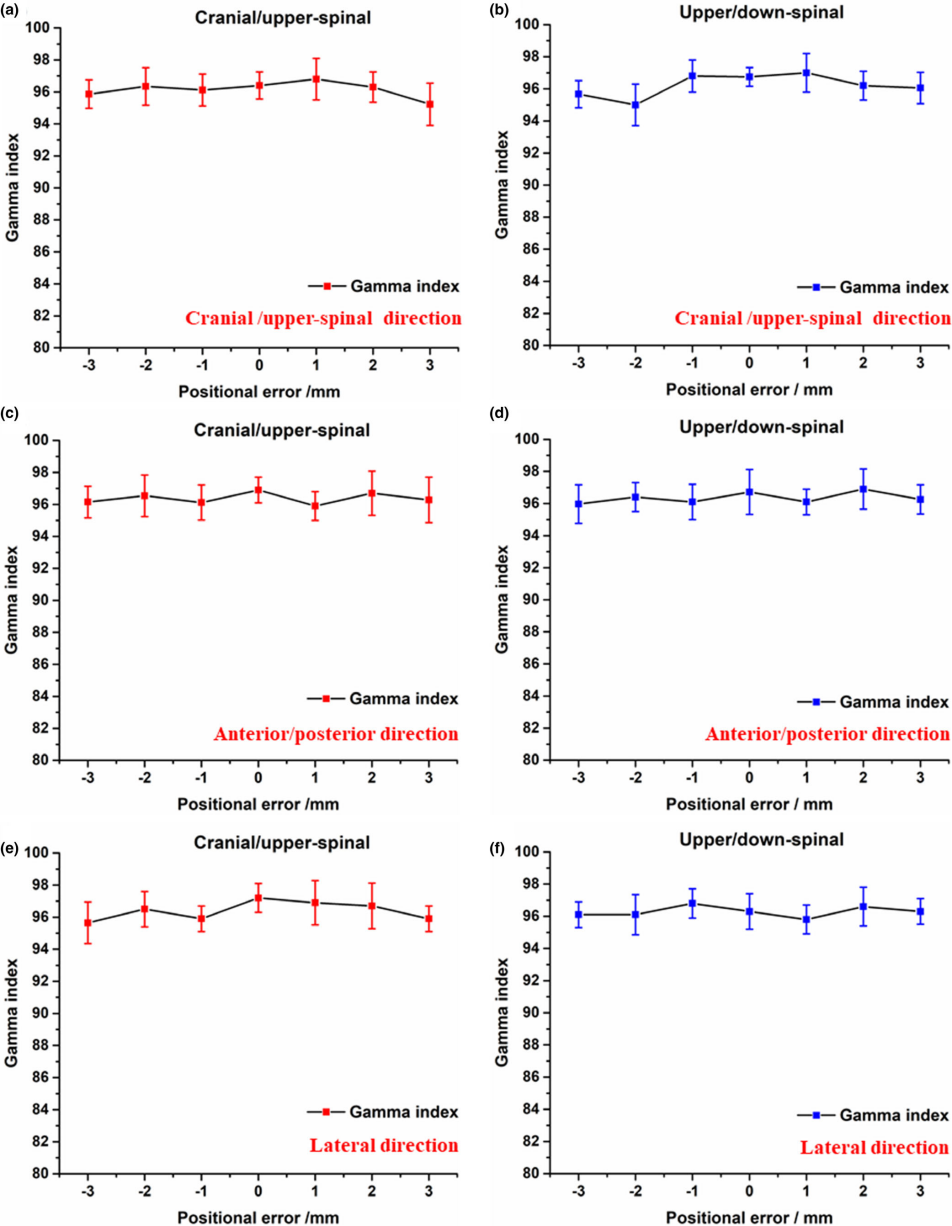
Dosimetric verification results with ArcCHECK; a), c), and e) the mean %GPs in the junctions between cranial/upper‐spinal field in the craniocaudal direction, anterior/posterior direction, and lateral direction; b), d), and f) the mean %GPs in the junctions between upper/lower‐spinal field in the craniocaudal direction, anterior/posterior direction, and lateral direction.

**Fig. 5:**
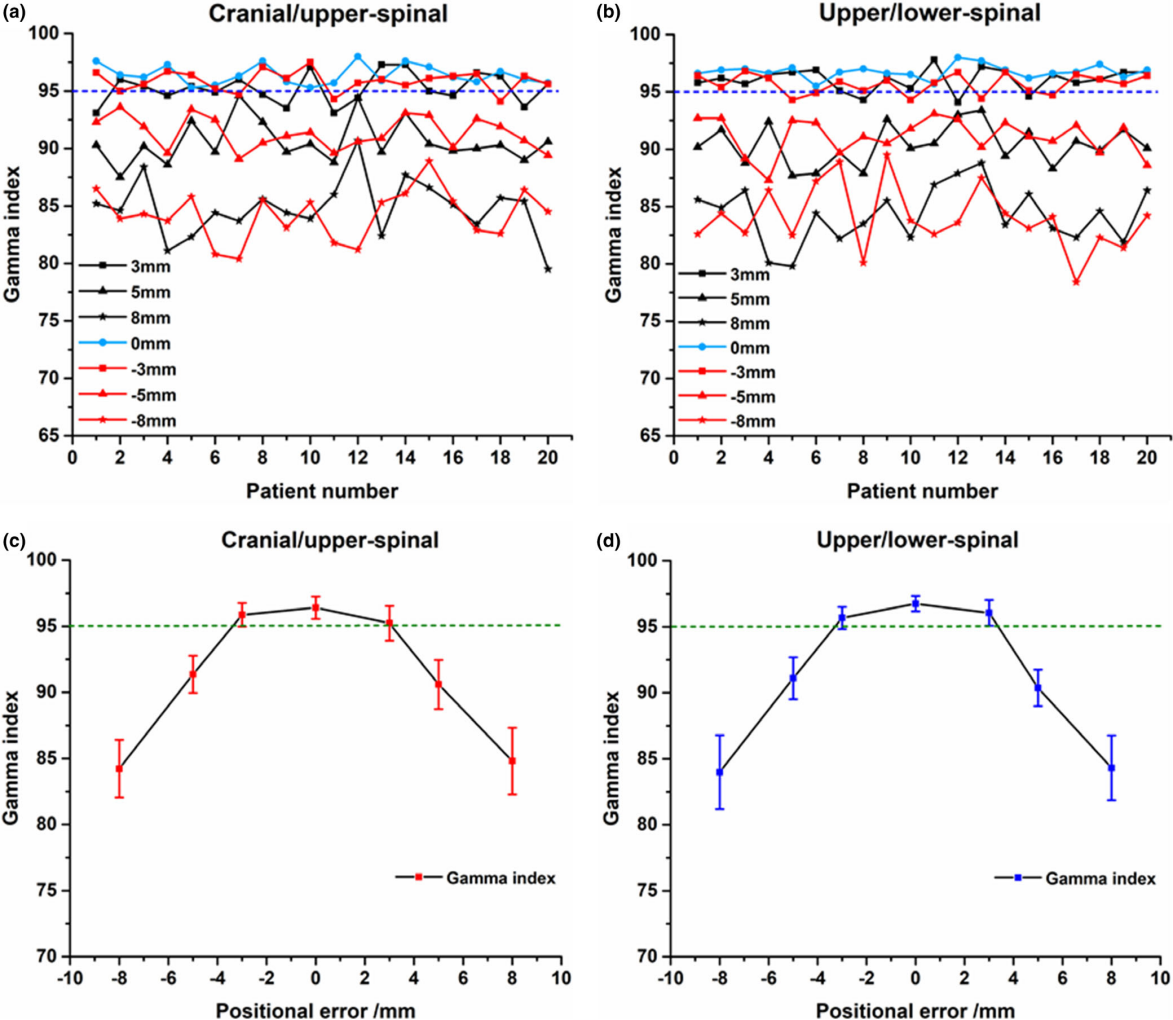
Dosimetric verification results with ArcCHECK; a) and b) the measured percentage gamma passing rates (%GPs) in the junctions between cranial/upper‐spinal field and upper/lower‐spinal field; c) and d) the mean %GPs in the junctions between cranial/upper‐spinal field and upper/lower‐spinal field.

Dosimetric verification results with ArcCHECK are presented in Fig. 4 and Fig. 5. Figures 4(a), 4(c), and 4(e) showed the mean %GPs in the junctions between cranial/upper‐spinal field in the craniocaudal direction, anterior/posterior direction, and lateral direction. Figures 4(b), 4(d), and 4(f) showed the mean %GPs in the junctions between upper/lower‐spinal field in the craniocaudal direction, anterior/posterior direction, and lateral direction. The mean %GPs in the junctions between cranial/upper‐spinal field and upper/lower‐spinal field were around 95% in case of the setup errors within 0–3 mm in the three orthogonal directions. Figures 5(a) and 5(b) showed the measured %GPs for all the 20 patients in the junctions between cranial/upper‐spinal field and upper/lower‐spinal field with different positional errors in the craniocaudal direction. %GPs for ± 3 mm positional errors were around 95%. %GPs decreased with the increase of positional errors. As further shown in [Figure 5(c) and 5(d)], the mean %GPs were around 90% and 85% for positional errors of ± 5 mm and ± 8 mm, respectively.

**Fig. 6:**
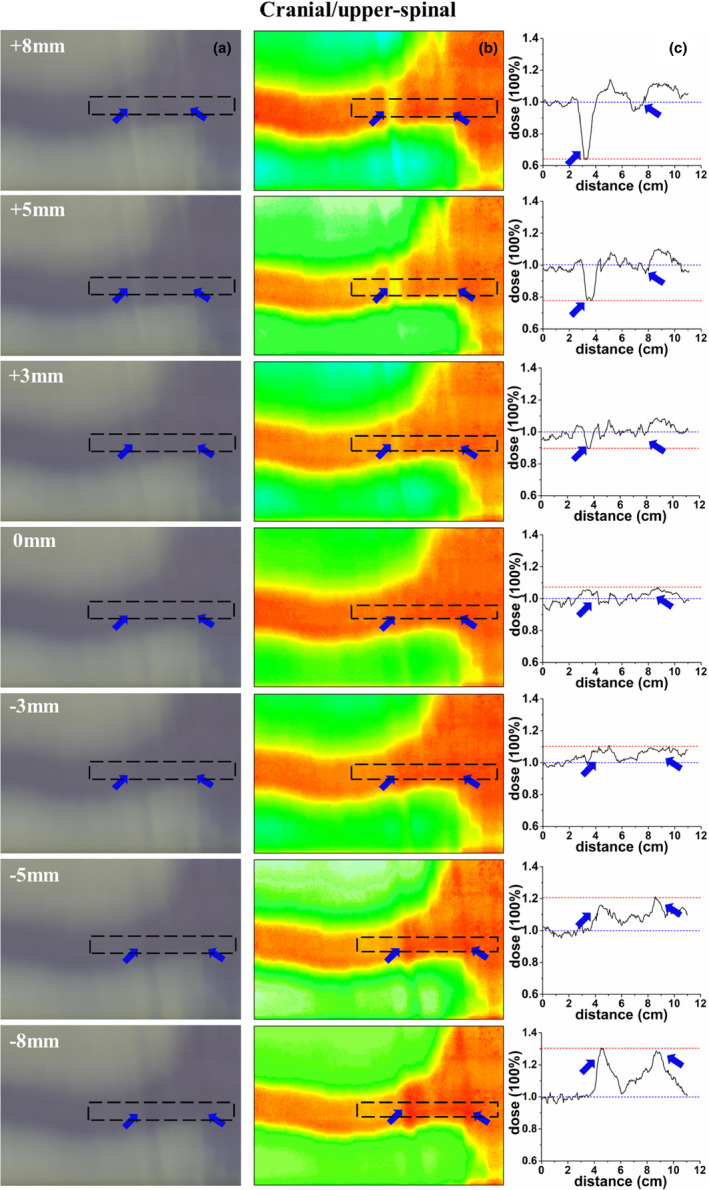
Verification of the cranial/upper‐spinal field junction; (a) an irradiated radiochromic film, (b) dose distribution of the radiochromic film, (c) dose profile along the dotted line, the dose profile was normalized to the prescription dose, and the blue arrow represent the hot/cold peaks of dose in the figures.

**Fig. 7:**
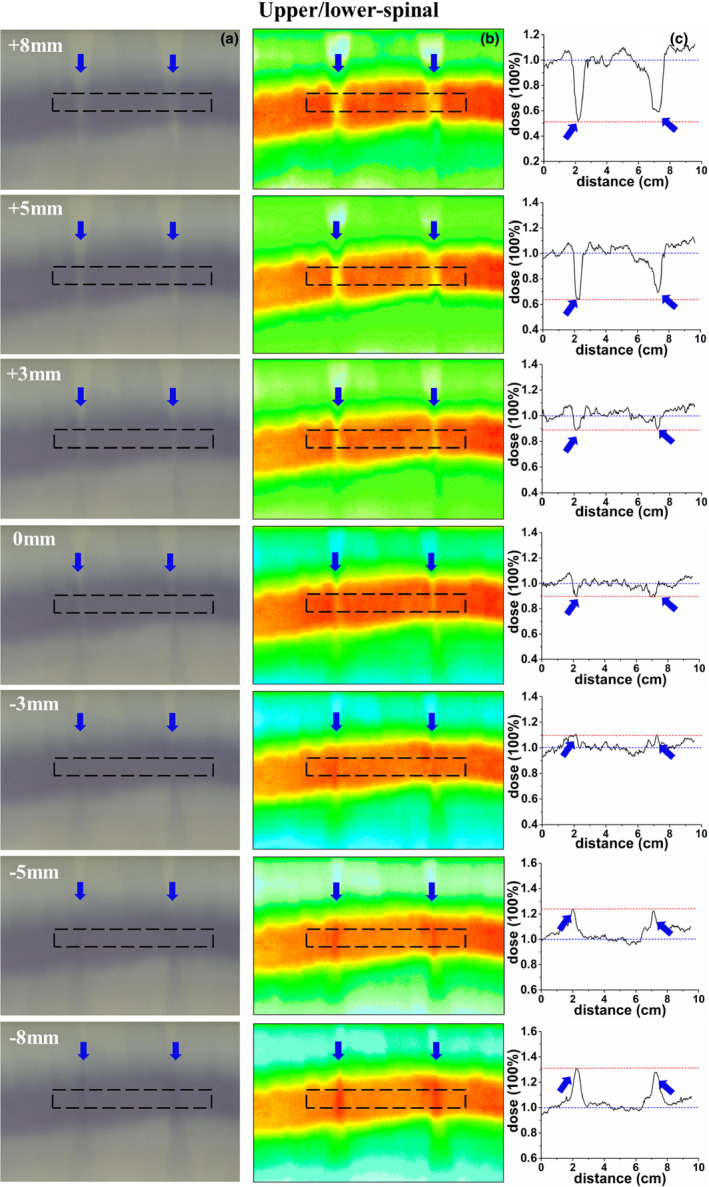
Verification of the upper/lower‐spinal field junction; (a) an irradiated radiochromic film, (b) dose distribution of the radiochromic film, (c) dose profile along the dotted line, the dose profile was normalized to the prescription dose, and the blue arrow represent the hot/cold peaks of dose in the figures.

The dosimetric verification results with EBT3 films are presented in Fig. 6 and Fig. 7. The gray scale films, hotmaps of dose distribution, and the dose profile across the field overlap region in the cranial/upper‐spinal field junction are shown in [Figs. 6(a)–6(c)], respectively. The gray scale films, hotmaps of dose distribution, and the dose profile across the field overlap region in the upper/lower‐spinal field junction are shown in [Figs. 7(a)–7(c)], respectively. As shown in Figures 6c and 7c, the dose profile was normalized to the prescription dose, and a positioning error up to ± 3 mm can result in a ± 10% change in dose distributions for cranial/upper‐spinal field junction and upper/lower‐spinal field junction, and this value increases to ± 35% in the case of a 5 mm positional shift, and increases to ± 50% in case of a 8 mm positional shift. Clear cold area and hot area were observed with the increase of introduced errors as shown in the figures.

## 4 Discussion

The dosimetric impact of positional errors on VMAT CSI was investigated by measuring the %GPs and the dosimetric distributions in the junction areas between the cranial/upper‐spinal field and the upper/lower‐spinal field with ArcCHECK and EBT3 films. No severe impact of normal positional errors on the delivery of multi‐isocenter VMAT CSI was observed.

The idea of irradiating the whole central nervous system for patients with cerebellar medulloblastoma was firstly advanced by Dr. Edith Paterson.^22^ The challenge along with this CSI technique for whole central nervous system is the extreme long target volume. As shown in Table 1, the PTV length for the enrolled patients in this study is around 50–80 cm. The maximum treatment field of linac‐based MLC is usually around 40 cm. Two to three isocenters were commonly required during CSI with modern IMRT and VMAT techniques.^7^, ^19^ Three‐isocenter VMAT plans were generated in this study to cover the brain and spinal as one PTV.

The purpose of treating the whole nerve system as one PTV is to decrease the dose inhomogeneity at the field junctions and to smooth the dose distribution with the intrinsic characteristics of the inverse optimization of VMAT.^4^, ^5^ Previous studies also reported that it is feasible to use overlapping VMAT fields in the treatment of long volumes of PTV by optimizing the overlapping fields concurrently without explicitly controlling the junction dose.^9^, ^21^ However, more strict requirement on time and effort during optimization and plan setup was required.

Dosimetric verification with ArcCHECK and EBT3 films in this study indicated that setup errors less than 3 mm have little impact on the VMAT delivery. Similarly, Sarkar et al. reported that VMAT technique is insensitive to longitudinal setup errors (1–3 mm) because of the existence of low dose gradients at the junction between fields.^16^ When the setup error was greater than 5 mm, lower %GPs and cold or hot junctions were observed in this study. However, even when the positional error reached 8 mm, the %GPs only had a minor degradation of around 85%. Similar results were demonstrated by Meyer et al., in which they simulated the isocenter shifting by 1, 2, 5, and 10 mm and concluded that isocenter shifting should be minimized, but the treatment plan accuracy will not be deteriorated even when larger errors of 5–10 mm were simulated.^20^ In general clinical practice, the setup errors are usually around 1–3 mm, especially for the application of modern IMRT and VMAT techniques, and high accuracy of setup error is a precondition.^23^

## 5 Conclusions

The dosimetric verification with intentionally introduced setup errors demonstrated that positional errors less than 3 mm have little impact on VMAT CSI, although setup errors should be minimized during practice. Relying on the inverse optimization of VMAT to smooth the dose distribution in the junction areas is feasible for CSI.

## Conflict of interests

The authors have declared that no competing interest exists.
